# Expression of immune-related genes as prognostic biomarkers for the assessment of osteosarcoma clinical outcomes

**DOI:** 10.1038/s41598-021-03677-y

**Published:** 2021-12-16

**Authors:** Junjie Guo, Xiaoyang Li, Shen Shen, Xuejian Wu

**Affiliations:** 1 Department of Orthopedics, The First Affiliated Hospital of Zhengzhou University, Zhengzhou, 450052 People’s Republic of China; 2 Department of Orthopedics, National Cancer Center/Cancer Hospital, Chinese Academy of Medical Sciences and Peking Union Medical College, Beijing, 100021 People’s Republic of China; 3 Gene Hospital of Henan Province, Precision Medicine Center, The First Affiliated Hospital of Zhengzhou University, Zhengzhou, 450052 People’s Republic of China

**Keywords:** Cancer, Sarcoma, Tumour biomarkers

## Abstract

Cancer immunotherapy is a promising therapeutic approach, but the prognostic value of immune-related genes in osteosarcoma (OS) is unknown. Here, Target-OS RNA-seq data were analyzed to detect differentially expressed genes (DEGs) between OS subgroups, followed by functional enrichment analysis. Cox proportional risk regression was performed for each immune-related gene, and a risk score model to predict the prognosis of patients with OS was constructed. The risk scores were calculated using the risk signature to divide the training set into high-risk and low-risk groups, and validation was performed with GSE21257. We identified two immune-associated clusters, C1 and C2. C1 was closely related to immunity, and the immune score was significantly higher in C1 than in C2. Furthermore, we validated 6 immune cell hub genes related to the prognosis of OS: CD8A, KIR2DL1, CD79A, APBB1IP, GAL, and PLD3. Survival analysis revealed that the prognosis of the high-risk group was significantly worse than that of the low-risk group. We also explored whether the 6-gene prognostic risk model was effective for survival prediction. In conclusion, the constructed a risk score model based on immune-related genes and the survival of patients with OS could be a potential tool for targeted therapy.

## Introduction

Osteosarcoma (OS) is the most common primary malignant bone tumor in children and young adults and has a high incidence of fatality^1^. The application of neoadjuvant chemotherapy significantly increased the survival rate to 60% in the 1980s, but it has since plateaued^2^. Other conventional strategies, including surgery and radiotherapy, offer substantial benefit to eliminate primary tumors, but disease relapse is still a commonly encountered problem that results from residual malignant cells and/or tumor metastases^3,4^. In such cases, effective alternative treatment approaches for OS treatment are warranted.

Cancer immunotherapy has become an established pillar of cancer treatment, improving the clinical outcomes of patients with a broad variety of malignancies^5,6^. The two main tools in the cancer immunotherapy field are chimeric antigen receptor (CAR) T cells and checkpoint inhibitors (CPIs)^6,7^. Bone consists of highly specialized cells of the immune system, and bone homeostasis is maintained by many immune signaling pathways^8^. The immune environment of OS consists of many cell subpopulations, such as T lymphocytes, macrophages, B lymphocytes and mast cells. Treatments in combination with immune cell therapy have been reported to substantially improve clinical outcomes^9^. Immunotherapy activates immune cells targeting attacked cancer cells^10^. As an immune checkpoint inhibitor, PD-1 increases the number of tumor-infiltrating T lymphocytes while activating depleted CD8 T cells.

Recently, the tumor microenvironment (TME), including innate and adaptive immunity, has been well studied. The TME has important effects on the tumorigenesis and development of OS. Changes in the TME and associated regulatory mechanisms are current research hotspots. Various studies have proven that immune-associated genes have significant prognostic value for predicting the clinical outcome of OS^11–13^. Zhang et al.^14^ illustrated that osteosarcoma patients with high immune scores had good prognosis and significantly lower expression levels of naive B cells and M0 macrophages. Cytotoxic T cell lymphocyte antigen 4 (CTLA4) and CD40 have been reported to break immune tolerance and exhibit antitumor capability in OS^15^. Scott et al.^16^ developed a novel score system based on the expression of genes in clusters in OS tumor samples and suggested that immune cell presence was significantly associated with good prognosis. However, additional challenges remain, and further scrutiny in cancer immunology and immunotherapies in OS is required.

In this study, we constructed risk score models based on a bioinformatics algorithm, utilized OS cohorts to predict clinical outcomes, and evaluated and validated the relationship between immune-related genes and associated pathways. We aimed to uncover the potential role of immune-related genes and associated pathways in OS.

## Results

### Subgroup classification and analysis of immune cell infiltrates and OS clinical characteristics

**Figure 1:**
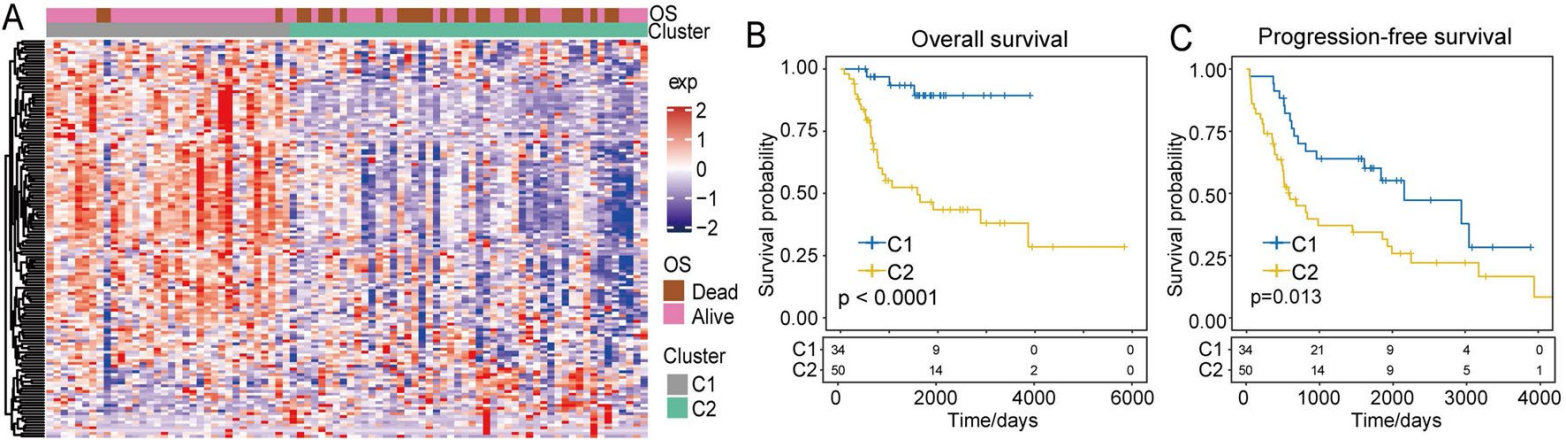
Landscape of immune cell-related gene expression and subtype prognosis. (**A**) Heatmap showing immune cell-related gene expression in the different subtypes. (**B**) Overall survival for the C1 and C2 subtypes. (**C**) Progression-free survival for the C1 and C2 subtypes.

To identify immune system-related hub genes, we evaluated the expression of 804 immune system-related genes and identified 142 of them as being closely related to OS prognosis. The NMF algorithm was used for this analysis (*k* = 2–10), and these 142 genes were classified into C1 and C2 subgroups. The C1 subgroup had higher expression levels of immune system-related genes and fewer deaths. However, C2 showed a lower immune gene expression level and a high frequency of patient death (Fig. 1A). Kaplan–Meier curves were used to assess prognoses between the two groups, and the analysis indicated that the C1 subgroup had a shorter overall survival rate than the C2 subgroup (*p* < 0.0001, Fig. 1B). The C1 subgroup also showed a shorter prognosis-free survival rate than the C2 subgroup (*p* = 0.013, Fig. 1C).

### Functional analysis of DEGs

**Figure 2:**
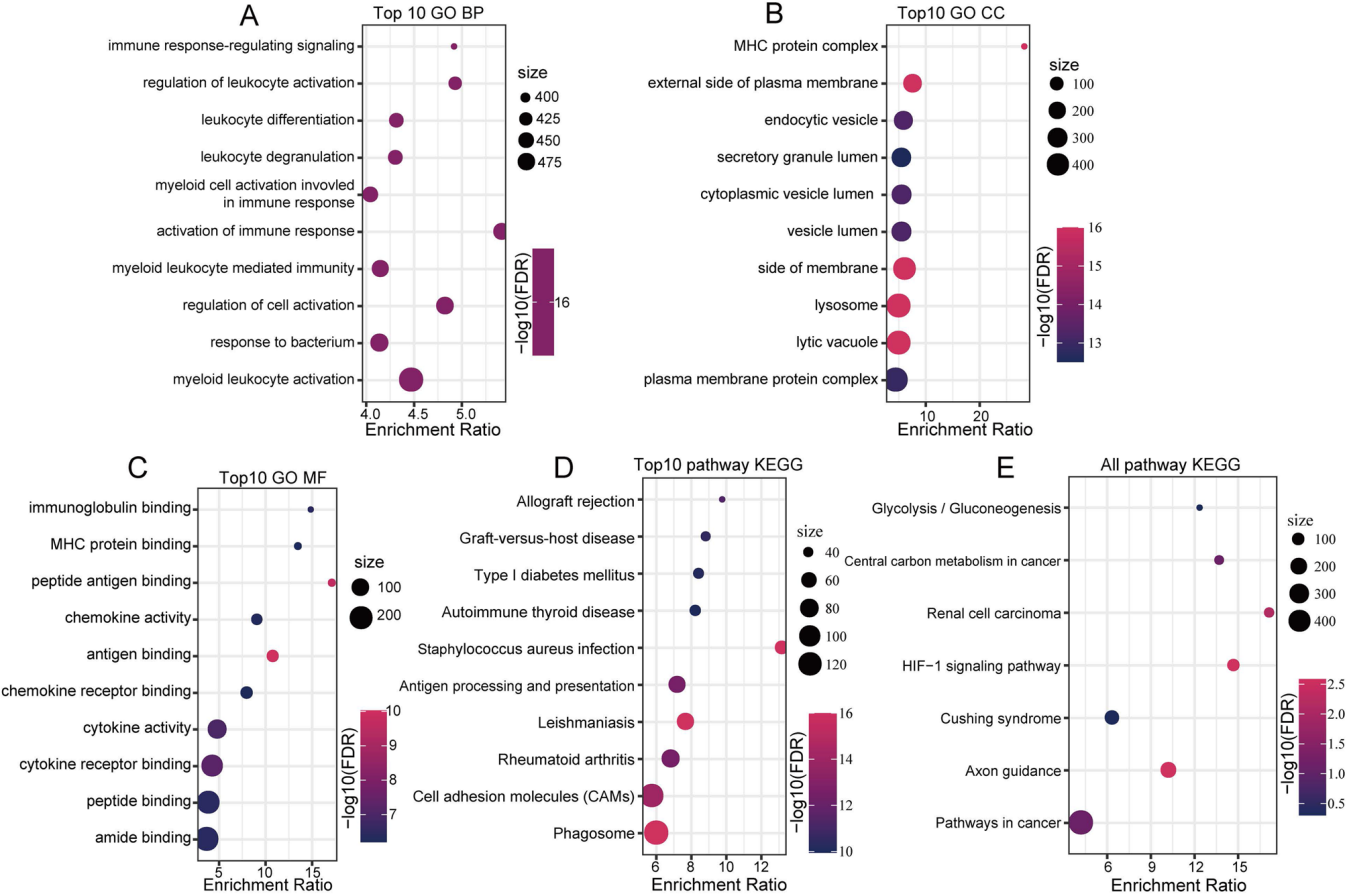
Pathway annotations for subtype-related differentially expressed genes (DEGs). (**A**) Pathway enrichment analysis of the upregulated DEGs using BP annotation. (**B**) Pathway enrichment analysis of the upregulated DEGs using CC annotation. (**C**) Pathway enrichment analysis of the upregulated DEGs using MF annotation. (**D**) Pathway enrichment analysis and annotation of the upregulated DEGs via KEGG analysis. (**E**) Pathway enrichment analysis and annotation of the downregulated DEGs via KEGG analysis.

To further investigate the biological functional pathways of the immune-related genes, GO and KEGG analyses were applied in this study. The top 10 significantly enriched biological process (BP) terms were related to myeloid leukocyte activation (Fig. 2A). The most enriched cellular component (CC) terms were plasma membrane protein complex, lytic vacuole, lysosome, side of membrane, vesicle lumen, cytoplasmic vesicle lumen, endocytic vesicle, and external side of plasma membrane (Fig. 2B). The most enriched molecular function (MF) terms included amide binding, protein binding, cytokine receptor binding, and cytokine activity (Fig. 2C). The KEGG enrichment analysis showed significant enrichment of the pathway terms phagosome, cell adhesion molecules (CAMs), rheumatoid arthritis, and leishmaniasis (Fig. 2D). The most significantly enriched KEGG pathways included pathways involved in cancer, axon guidance, and Cushing syndrome (Fig. 2E).

### Significant differences in immune-related characteristics between the two clusters

**Figure 3:**
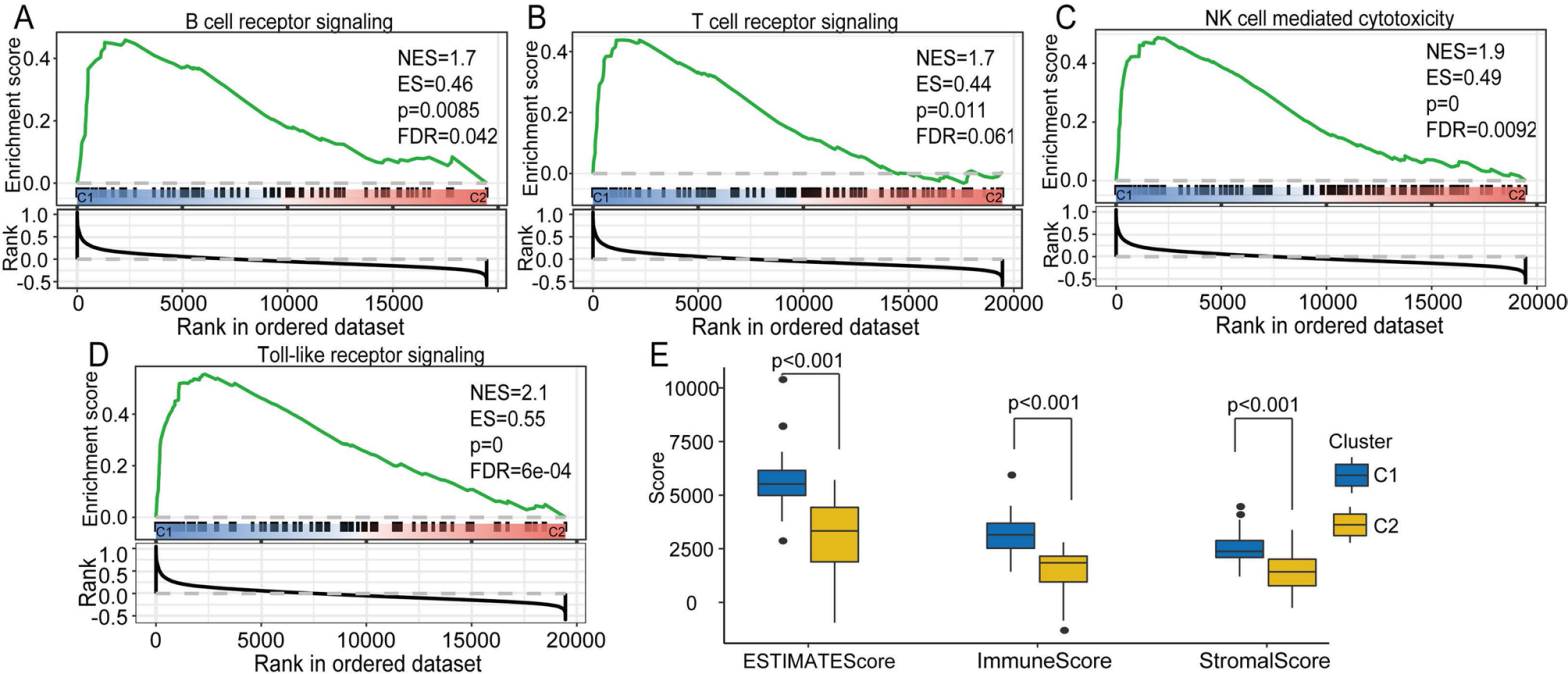
Immune system-related pathway analyses and evaluation by group subtype. (**A**–**D**) KEGG analysis of immune system-related pathways showed enrichment of the B cell receptor signaling pathway, the T cell receptor signaling pathway, natural killer cell-mediated cytotoxicity pathways, and the Toll-like receptor signaling pathway. (**E**) Comparisons of stromal score, immune score, and estimate score between the C1 and C2 subgroups.

We obtained further insights into the differences in the immune and matrix scores between the two molecular subtypes. Our research demonstrated that there were obvious differences between C1 and C2 in the stromal score**,** immune score, and estimate score. The results indicated that immune cell-related pathways (the B cell receptor signaling pathway (ES = 0.46, *p* = 0.009, FDR = 0.42, Fig. 3A), the T cell receptor signaling pathway (ES = 0.44, *p* = 0.011, FDR = 0.061, Fig. 3B), natural killer (NK) cell-mediated cytotoxicity pathways (ES = 0.49, *p* = 0, FDR = 0.009, Fig. 3C), and the Toll-like receptor signaling pathway (ES = 0.55, *p* = 0, FDR = 6e−04, Fig. 3D) were enriched in the C1 subtype. These results demonstrated that the C1 subtype was closely associated with immune cell receptors and cytotoxic activities. To gain further insight into the differences in the immune score and matrix score between the two subtypes, we determined the significant differences between C1 and C2. The estimate score in C1 was remarkably higher than that in C2 (*p* < 0.001). The immune scores were also significantly higher in the C1 subgroup than in the C2 subgroup (*p* < 0.001). The C1 subgroup demonstrated higher immune scores than the C2 subgroup (*p* < 0.001, Fig. 3E). These results demonstrated that the overall immune score of the C1 subtype was significantly higher than that in the C2 subtype.

### Construction of a prognostic risk model based on immune-associated genes

**Figure 4:**
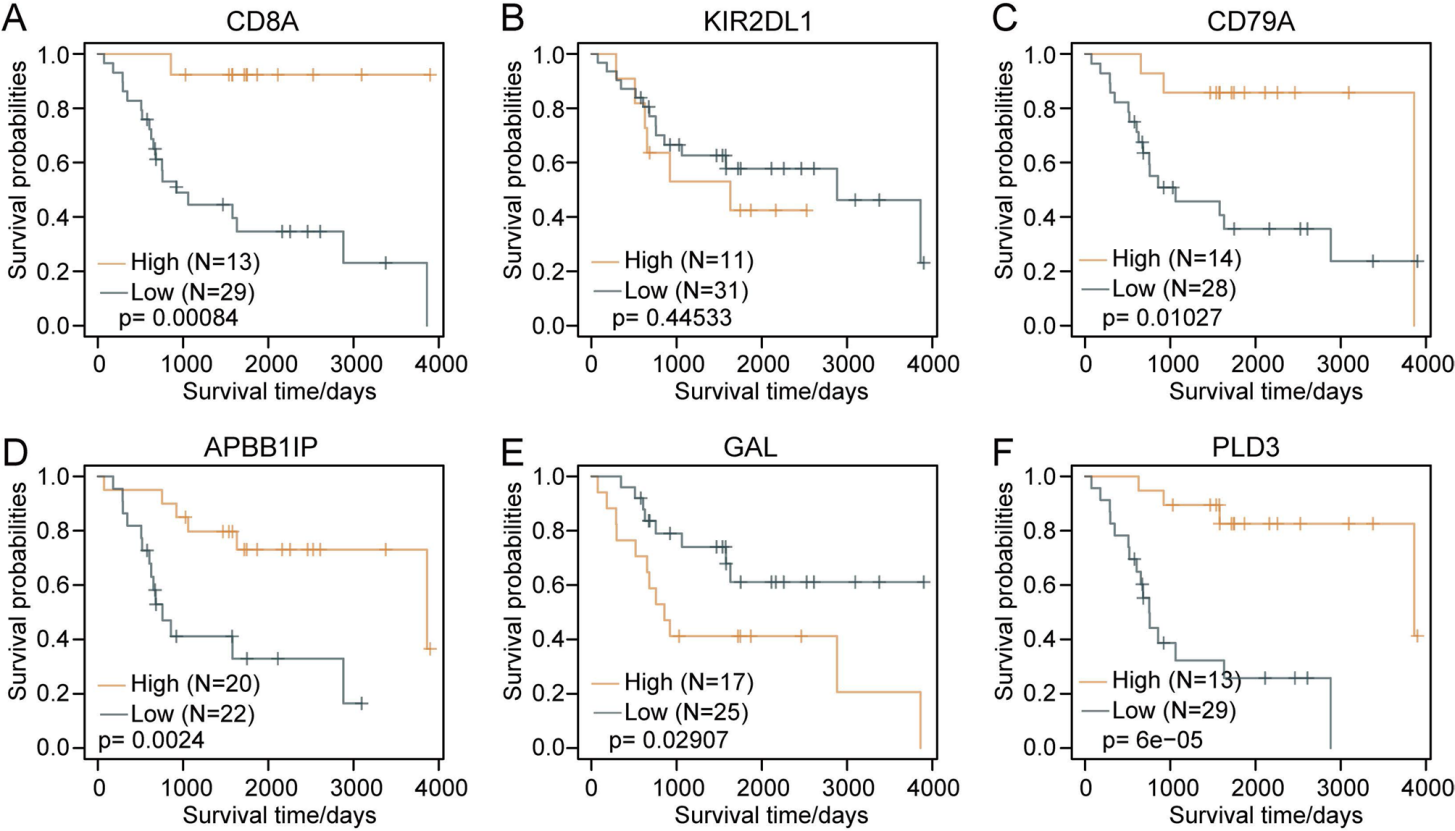
Kaplan–Meier curves for the 6 genes (in the TARGET training cohort). (**A**) Kaplan–Meier curve for the CD8A gene. (**B**) Kaplan–Meier curve for the KIR2DL1 gene. (**C**) Kaplan–Meier curve for the CD79A gene. (**D**) Kaplan–Meier curve for the APBB1IP gene. (**E**) Kaplan–Meier curve for the GAL gene. (**F**) Kaplan–Meier curve for the PLD3 gene.

We investigated immune cell genes related to the prognosis of OS. We obtained 6 hub genes, CD8A, KIR2DL1, CD79A, APBB1IP, GAL and PLD3. Then, we generated Kaplan–Meier curves of prognosis for the 6 genes. This result suggested that in addition to the KIR2DL1 gene, the remaining 5 genes could significantly divide the Target-OS training set samples into high- and low-risk groups. We performed Kaplan–Meier analyses of the six genes. The results showed that the high CD8A expression subgroup had better survival probabilities than the low CD8A expression subgroup (*p* = 0.00084, Fig. 4A). The higher CD79A subgroup showed better prognoses than the low CD79A expression subgroup (*p* = 0.01027, Fig. 4C). In contrast, the APBB1IP overexpression group had significantly better prognosis than the low APBB1IP expression group (*p* = 0.0024, Fig. 4D). The high PLD3 expression subgroup had remarkably better prognosis than the low PLD3 expression group (*p* = 6e−05, Fig. 4F). In contrast, the high GAL expression subgroup had poor prognosis compared to the low GAL expression subgroup (*p* = 0.02907, Fig. 4E). In addition, there was no significant difference between the different KIR2DL1 expression subgroups (*p* = 0.44533, Fig. 4B). Overall, these results demonstrated that these six genes were significantly associated with prognosis.

### Construction and evaluation of risk models including the six immune-related genes

**Figure 5:**
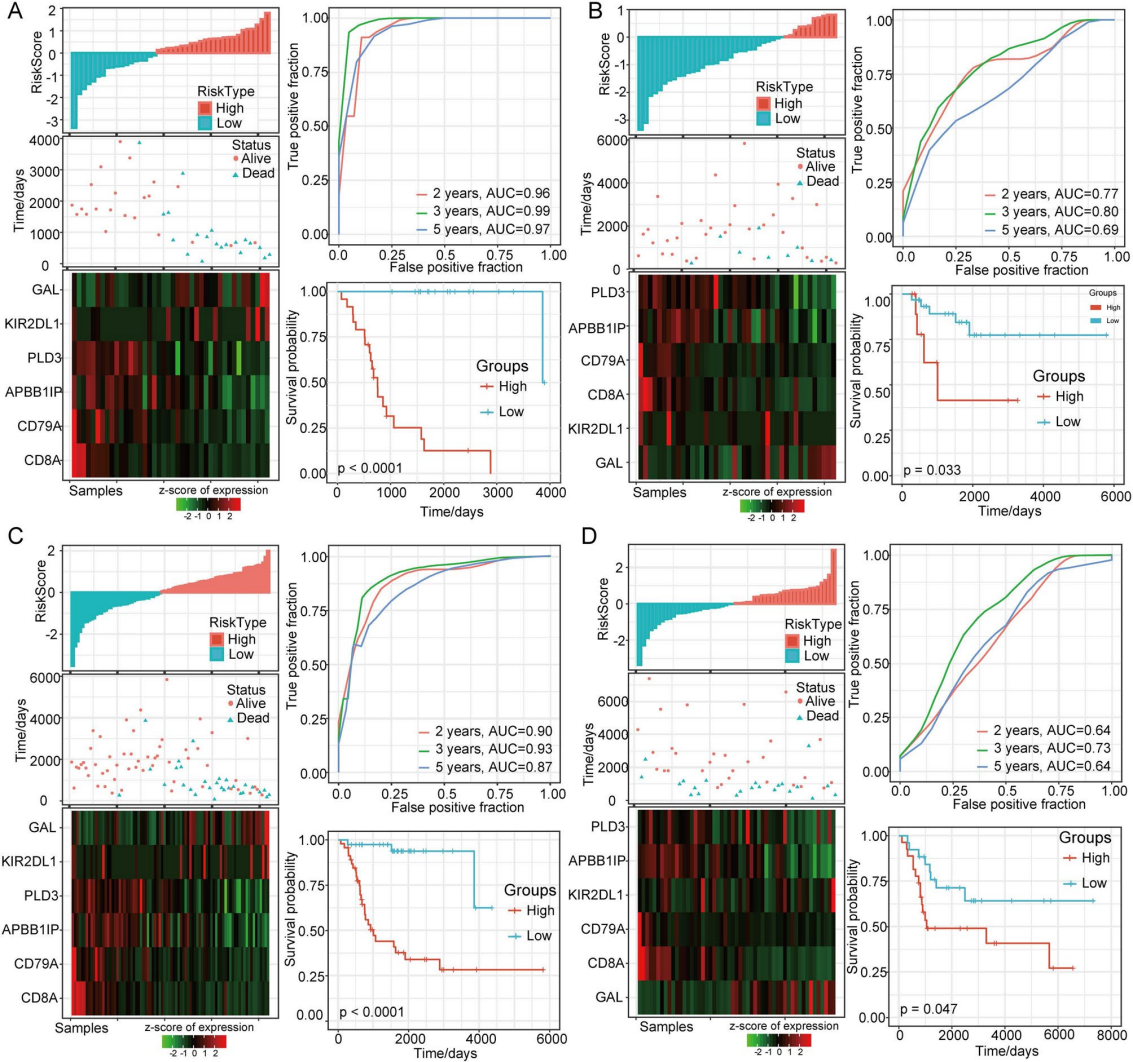
Construction and assessment of the risk model with the OS training set and validation datasets. (**A**) Construction and evaluation of the risk model with the OS training set. (**B**) Verification of the robustness of the 6-gene signature with the internal OS validation dataset. (**C**) Verification of the robustness of the 6-gene signature with the internal OS dataset. (**D**) Verification of the robustness of the 6-gene signature with the external GSE21257 set.

To determine the prognosis-predicting ability of the six hub genes in OS, ROC curve analysis was performed. The distribution of risk scores suggested that the overall survival of OS patients with high risk scores was significantly lower than that of patients with low risk scores, indicating that the high risk score patients had poor prognosis. Assessment of the prognostic risk associated with the 6 DEGs indicated that high expression of GAL and KIR2DL1 was closely correlated with poor outcome and could act as risk factor, while upregulation of CD8A, CD79A, APBB1IP and PLD3 was significantly associated with good outcome and could act as a protective factor. Furthermore, ROC analysis of the ability of the risk score to predict prognosis was conducted, and the classification efficiency for 2-, 3- and 5-year outcomes was assessed. The model had a high AUC value (Fig. 5A). Finally, the samples with a risk score greater than 0 after Z-score standardization were divided into a high-risk group, and those with a risk score less than 0 were divided into a low-risk group. Twenty-four of the samples were classified as high risk, and 18 samples were classified as low risk. Survival analysis showed that the prognosis of the high-risk group was significantly worse than that of the low-risk group. According to the expression level of the samples, the risk score of each sample was calculated, the risk score distribution of the samples was determined, and survival analysis and time-dependent ROC curve analysis were performed (Fig. 5B). GAL and KIR2DL1 were identified as risk factors, while CD8A, CD79A, APBB1IP and PLD3 were identified as protective factors. The AUC of the model was higher than that of previously established models. Kaplan–Meier survival analysis showed that the prognosis of patients in the high-risk group was significantly worse than that of patients in the low-risk group (*p* = 0.033). Similar conclusions were reached in the entire Target-OS dataset (Fig. 5C) and the external independent validation dataset GSE21257 (Fig. 5D).

### Prognosis risk model and clinical characteristics

**Figure 6:**
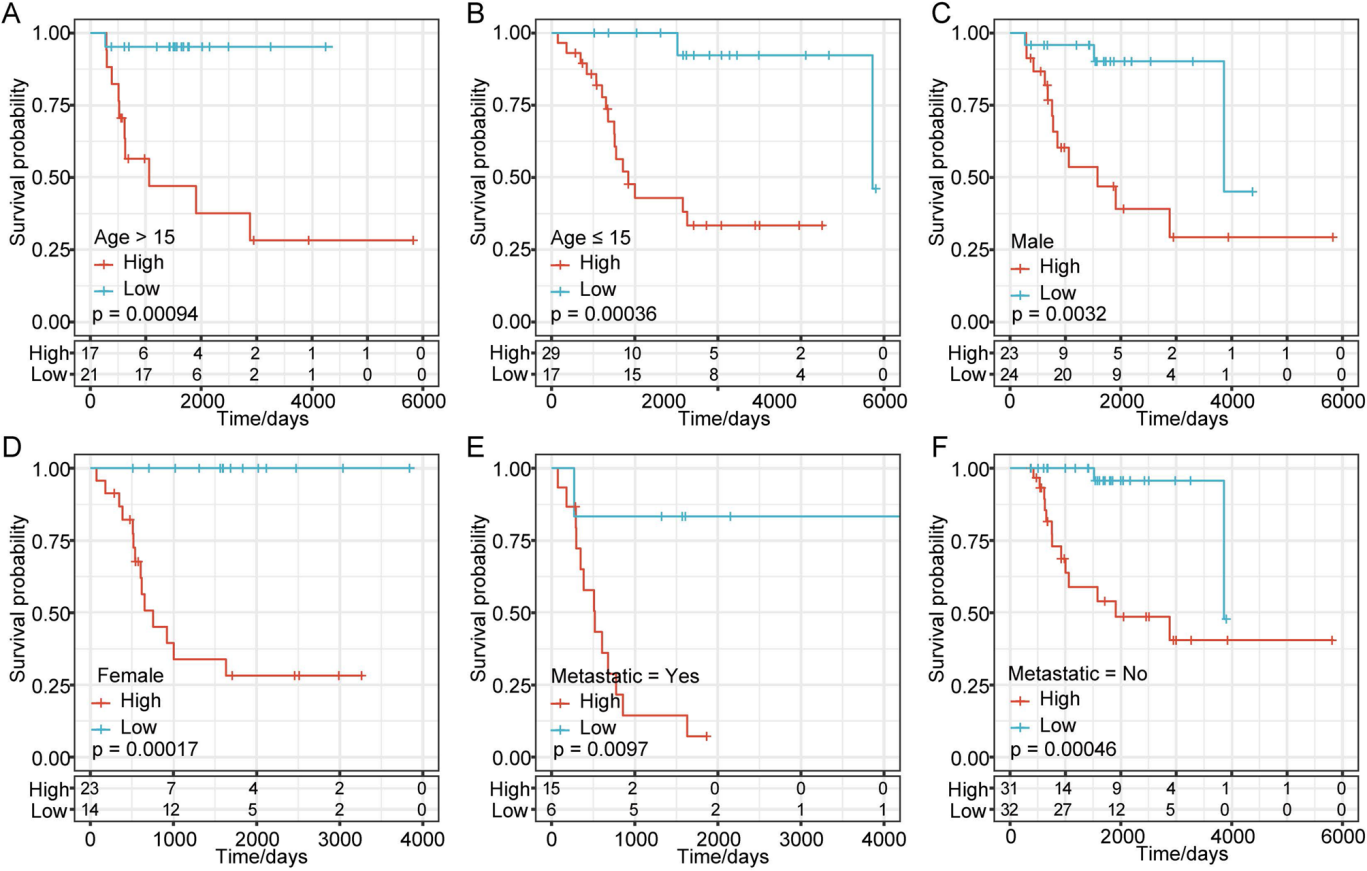
Kaplan–Meier curves for clinical characteristics. (**A**) Kaplan–Meier curve based on age > 15 years. (**B**) Kaplan–Meier curve based on age ≤ 15 years. (**C**) Kaplan–Meier curve of the male group. (**D**) Kaplan–Meier curves of the female group. (**E**) Kaplan–Meier curve of the group with metastasis. (**F**) Kaplan–Meier curve of the group without metastasis.

To further investigate the clinical features of these hub genes, the risk score and clinical characteristics such as age, sex, and metastasis were assessed and found to be independent but complementary prognostic factors. We found that the risk score calculated based on the 6-gene risk model could significantly distinguish high- and low-risk groups in each subgroup based on age, sex and metastasis. Furthermore, the Kaplan–Meier curve based on age > 15 years demonstrated significant differences between the high-risk and low-risk groups (*p* = 0.00094, Fig. 6A). The Kaplan–Meier curve based on age ≤ 15 years exhibited obvious differences between the high-risk and low-risk groups (*p* = 0.00036, Fig. 6B). Similarly, marked differences between the high-risk and low-risk groups were also illustrated in the sex (Fig. 6C,D) and tumor metastasis (Fig. 6E,F)-based subgroups. This result suggested that this model has good predictive ability in patients with different clinical features.

### Relationship between the risk score and biological function

**Figure 7:**
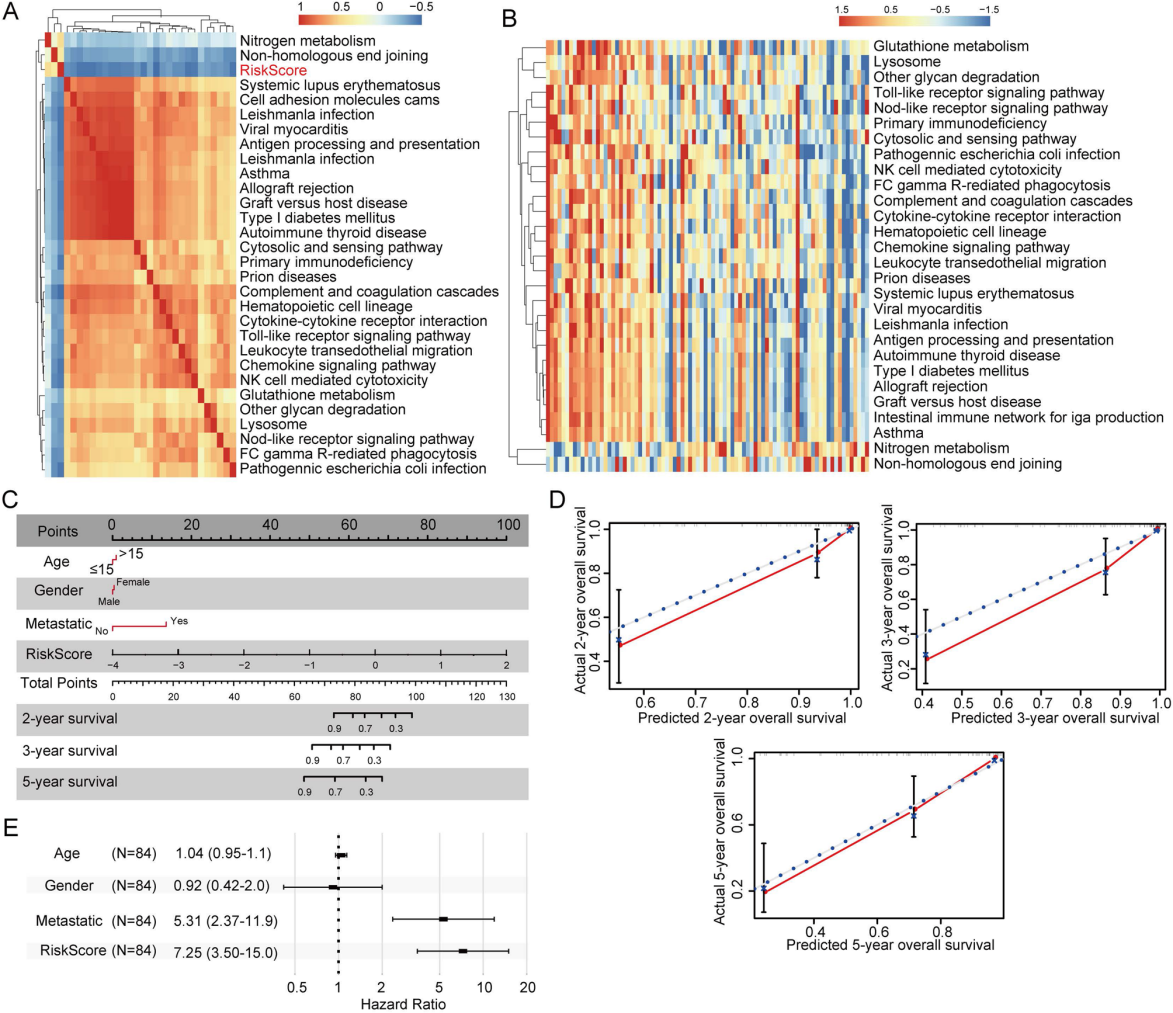
Relationship between the risk score and gene-related pathways and clinical features. (**A**) The relationship between the risk score and pathways as assessed with GSEA. (**B**) KEGG pathway analysis of the risk score. (**C**) Line chart of the risk score and clinical features. (**D**) Nomogram with correction based on the survival rate. (**E**) Forest plot of the risk score and clinical features. GSEA, gene set enrichment analysis.

To investigate the relationship between the risk score and biological functions in different samples, the corresponding gene expression profiles of the samples were analyzed by ssGSEA. The scores of different functions of each sample were calculated, and the correlation between these function scores and the risk score was further calculated. Functions with correlations greater than 0.35 were selected (Fig. 7A). Twenty-seven functions were negatively correlated with the risk score, while the other two were positively correlated with the risk score. KEGG pathways were selected for clustering analysis based on their enrichment scores (Fig. 7B). Nitrogen metabolism and nonhomologous end joining were among the 29 pathways increased in samples with an increased risk score. The Toll-like receptor signaling pathway, NK cell-mediated cytotoxicity pathways, and viral myocarditis-related metabolic pathways were decreased in samples with an increased risk score. These findings indicated that the dysregulation of these pathways is closely associated with the development of tumors. Based on the entire Target-OS dataset, a nomogram model including age, sex, metastasis, and the risk score was constructed (Fig. 7C). The risk score had the greatest influence on survival prediction. In addition, we tested the performance (Fig. 7D) for 2-, 3- and 5-year outcomes with histogram data, and the results showed that the method had good performance.

Clinical features, such as age, sex, and metastasis and the risk score were visualized in a forest plot (Fig. 7E). The risk score hazard ratio (HR) was approximately 7.25, which suggested that the prognostic risk model can be used as a prognostic risk factor. Furthermore, we performed univariate and multivariate Cox regression analyses to identify independent risk factors. We found that the risk score was significantly correlated with survival, and the corresponding multivariate Cox regression analysis also found that the risk score (HR = 7.248, 95%CI = 3.504–14.994, *p* < 0.001) was significantly correlated with survival. The above results show that our 6-gene signature has good predictive performance and clinical application value.

## Discussion

Immunotherapy has provided a new approach for the treatment of OS based on the exploration of the immune microenvironment^1^. Immunotherapy and gene therapy are novel treatments for OS^17^. Risk assessment and identification of immune gene-associated subtypes for determining the prognosis of OS cases need improvement. A large amount of evidence shows that the tumor microenvironment contributes to tumor differentiation, proliferation and distant metastasis^18,19^. Immune dysregulation has a close relationship with OS initiation and progression. Liu et al.^20^ proposed a risk score-based 14 immune-related genes that had good and stable sensitivity and specificity in predicting OS prognosis^21^. In this study, we also identified a six-immune-related gene (CD8A, KIR2DL1, CD79A, APBB1IP, GAL and PLD3) prognostic model that had good prognostic value in OS. Like Liu et al*.*^20^, we also applied LASSO Cox, AIC and stepdown regression. We performed LASSO analysis, which has obvious advantages in gene model construction. LASSO analysis allows analysis and processing of each independent variable simultaneously, which greatly increases the stability of the model.

Immune score assessment is very important. Zhang et al.^14^ established an estimate algorithm to assess the immune score of OS samples and classified them into high and low immune score groups. Liu et al.^20^ identified four pseudogene classifiers: ribosomal protein (RP) L11-551L14.1, RPL7AP28, RP4-706A16.3, and RP11-326A19.5, that served as overall survival markers in OS and were related to phenotype, immune, and DNA/RNA editing data. Receptor activator of nuclear factor-κB (NF-κB) ligand was also reported to participate in the genesis of OS^22,23^. Our results were consistent with previous studies. We analyzed the RNA-seq data of the Target-OS cohort and performed molecular subtype clustering based on immune cell-related genes. The samples were divided into two categories, C1 and C2, and the prognosis of the C1 subtype was better than that of the C2 subtype. Functional analysis revealed that samples of the C1 subtype were related to immunity, which indicated that the higher immune cell infiltration in C1 might enhance antitumor capability. Based on immune cell-related genes, we constructed a six-immune cell-related gene model for assessing prognostic risk including the following genes: CD8A, killer immunoglobulin-like receptor DL1 (KIR2DL1), CD79A, amyloid-β (A4) precursor protein-binding family B member 1 interacting protein (APBB1IP), GAL and phospholipase D3 (PLD3). These genes showed significant correlations with overall survival.

Li et al.^24^ found three key genes, 5-lipoxygenase activating protein (ALOX5AP), CD74 and CD32A, which were highly expressed in lungs and lymph nodes and could serve as OS metastasis biomarkers. Alpha-actinin-4 has been reported to enhance the proliferation and metastasis of OS, and its promotion of malignancy was validated to occur via the NF-κB pathway^25^. Researchers have identified variable KIR ligands in OS cell lines, and these cell lines had different susceptibilities to NK cell-mediated antitumor cytotoxicity^26^. In a dog model, researchers reported that high expression of the CD3 and CD8 genes in OS indicated better overall survival^27^. Ducharme et al.^28^ suggested that MYD88 and CD79B somatic mutations were common in the earliest cases of large B cell lymphoma, and these results might provide a strategy for early recommendation of first-line therapeutic strategies. In addition, APBB1IP is a signature gene that contributes to clinical gastric cancer (GC) diagnosis and early detection, and the PLD4 and PLD3 enzymes strongly affect inflammatory cytokine production via the degradation of nucleic acids, thus serving as important regulators of inflammatory disease^14^. In this study, we illustrated that the hub genes might directly or indirectly regulate immune cell function, thus affecting antitumor efficiency. This hypothesis still needs further validation.

Our model was validated with the Target-OS sample validation dataset, the Target-OS full dataset, and the independent validation dataset GSE21257, indicating the reliability of our model, which better predicted the risk of OS than did other previously established models. From the nomogram analysis results, the risk model had no correlation with clinical characteristics such as sex and age, indicating that these characteristics were independent. Immune-associated pathways have been suggested to have therapeutic potential across cancers^29^. In this analysis, we figured out 6 immune related genes model to predict OS clinical prognosis, and these 6 genes had been reported to play an important role in OS immune infiltration and prognosis. Wu et al.^21^ found that the immune features indicated different immune infiltration levels of OS, and suggested immunotherapeutic opportunities in OS patients. CD8A functioned as key immune activation and cytotoxicity gene in OS, which provided insight into potential biomarkers of response and resistance in OS immunotherapies^27,30^. KIR2DL1 acted as an important NK cell inhibitory function, which provided an important insight into the mechanism of inhibitory immune checkpoints in future immunotherapies^31^. Another study also validated that low-risk OS patients had significantly higher CD79A expression^32^. In addition, Cao et al.^33^ demonstrated that APBB1IP may act as a key to predicting OS prognosis and new biomarkers are of great significance for understanding the therapeutic targets of OS. Fan et al.^34^ clustered patients based on the immune cell infiltration content to identify a 9-prognostic gene signature gene model, including PLD3 for OS microenvironment exploration. Therefore, we observed that these 6-gene model might have good discrimination ability in predicting immune infiltration and clinical prognosis in OS. The gene model may also provide novel insights into the development of immunotherapies for OS.

However, this research has certain limitations. Through, we have confirmed that the 6-gene based on bioinformatic analysis, there is still no wet-lab experiment data backing up these 9 genes’ prognostic abilities and their roles in the immune infiltration. Accordingly, prospective studies are urgently needed to confirm our findings to aid in personalized clinical practice.

## Conclusions

In summary, based on the expression profiles of OS samples, we identified 6 hub genes. However, further extensive experiments are needed to confirm the results of this study. In addition, pathways associated with the hub genes are crucial in OS formation, progression and metastasis. These immune-associated genes and pathways might be biomarkers or targets for the diagnosis and treatment of OS.

## Methods

### Gene profiling data preprocessing

The expression profiles of 804 genes in OS patients were downloaded from the Target-OS database (Target-OS, https://ocg.cancer.gov/programs/target) on February 20, 2020. A total of 53 samples from GSE21257 chip data were obtained from the Gene Expression Omnibus (GEO, www. ncbi.nlm.nih.gov/geo) as an independent validation cohort. All pertinent clinical information, such as follow-up time, survival state, and gene expression levels in the 2 datasets, was available. The gene sets expressed by immune cells were obtained from relevant literature on immune cell research^35^, and genes listed at least 2 times were reserved, resulting in 864 total genes. Samples with insufficient or no clinical follow-up data were excluded from further analysis. After these preprocessing steps, we obtained 84 samples with 804 immune-associated genes from Target-OS and 53 patients from GSE21257 as the validation cohort.

### Immune-based classification and comparison

Univariate analysis was conducted via the Cox model to identify correlations of OS genes with the prognosis. The nonnegative matrix factorization (NMF) algorithm was applied to the BGM dataset of each motor behavior. A K of 2 to 10 was used to identify the optimal clusters: C1 and C2. Kaplan–Meier survival curves were plotted to show survival time. The R package was used to normalize the RNA-seq data and extract differentially expressed genes (DEGs). The DEGs were submitted to Gene Ontology (GO) and Kyoto Encyclopedia of Genes and Genomes (KEGG) enrichment (http://www.genome.jp/kegg/) analyses. These analyses were carried out separately on the upregulated and downregulated DEGs. Gene set enrichment analysis (GSEA) and pathway enrichment analyses were performed to further identify significantly enriched pathways, including the KEGG pathways, between C1 and C2. The immune score and stromal score of each sample were calculated in samples from both clusters with the R package ‘estimate’.

### Establishment of the prognostic risk model

The Target-OS dataset included the expression profiles of 804 immune cell-related genes. Eighty-four samples from the Target-OS dataset were divided into a training set and a validation set. To avoid random allocation bias affecting the stability of subsequent modeling, we use random group sampling to obtain 42 training set samples and 42 test set samples. Using the training set data and the R ‘survival’ package coxph function, a unilateral Cox proportional hazard regression model and multifactor risk analyses were performed for each immune-related gene and the survival data. The ‘glmnet’ package was used to perform the least absolute shrinkage and selection operator (LASSO) Cox regression, which performs regularization and selects variables simultaneously. Based on Akaike information criterion (AIC) and stepdown regression, minimal and appropriate models were obtained. We obtained 6 genes and performed corresponding survival analysis. Furthermore, the 6 genes were used to construct a risk model: Risk score = 8.549 * KIR2DL1 + 0.347 * GAL − 1.537 * CD8A − 1.1 * CD79A − 0.713 * APBB1IP − 1.159 * PLD3. Subsequently, the risk score distribution of the samples was determined. We used R software to perform time-dependent receiver operating characteristic (ROC) analysis of the risk score for 2-, 3-, and 5-year outcomes. Next, we calculated the Z-score of the risk score, and classified the samples whose risk score was greater than zero after the Z-score analysis into the high-risk group. Samples with a risk score less than zero were classified into the low-risk group, and the Kaplan–Meier survival curve was drawn.

### Risk model validation

Internal and external datasets were used to validate the robustness of the 6-gene signature. To establish the model, we calculated the risk score of patients in the Target-OS validation cohort and of patients in all the datasets. The method has been described previously. To verify the utility of the risk score in predicting the survival time and survival status of patients in the dataset, the expression of the 6 genes, the area under the ROC curve (AUC) values, and the 6-gene signature Kaplan–Meier survival curve distribution of the GSE21257 cohort were analyzed.

### Prognostic analysis of the risk model, clinical features and associated pathways

The risk score from the 6-gene signature and clinical characteristics of patients, such as age, sex, and metastasis, were assessed through Kaplan–Meier curve analysis. Single-sample GSEA (ssGSEA) was performed with the R software package ‘GSVA’, and each sample was analyzed. Based on the scores on different functions, we further calculated the correlation between these functions and the risk score, and a correlation greater than 0.35 was selected. We selected the corresponding KEGG pathways and performed clustering based on the results of their enrichment score analysis. We utilized the Target-OS database cohort, and the clinical features age, sex, and metastasis and the risk score were used to build a collinear map and a forest map, which clearly exhibited the overall statistical results and risk model results. Single-factor and multifactor analyses of the 6-gene signature and clinical overall survival were performed separately.

Ultimately, we selected three prognostic risk models: a 19-gene signature (Goh)^36^ 8-gene signature (Zhang)^26^ and 3-gene signature (Shi)^37^ were compared with our 6-gene model. To make the models comparable, we calculated the risk score of the Target-OS samples using the same method based on the corresponding genes in the 4 models, the Z-score, and the risk score, and divided the samples whose risk score was greater than zero and lower than the Z-score. The high-risk score group and the low-risk group were determined, and the difference in prognosis between the two groups of samples was validated. ROC and Kaplan–Meier curve analyses of the three models were performed, the results were compared, and the prognostic assessment capability of each model was compared.

### Statistical analysis

Continuous variables with a normal distribution are reported as the mean ± standard deviation (SD), while continuous variables with a skewed distribution are reported as the median (25th percentile to 75th percentile). Classification variables are reported as frequencies (scale). A t-test was used to compare continuous data with a normal distribution between two groups, and P values less than 0.05 were considered statistically significant. The difference between rates was tested by a chi-square test or Fisher’s exact test. Kaplan–Meier survival and log-rank test analyses were performed using the R ‘survival’ package. We used R software to performed time-dependent ROC analysis to determine the prognostic classification ability of the risk score.

## Data Availability

Human data was analyzed, all methods were performed in accordance with the relevant guidelines and regulations.
